# Modulation of the Inflammasome Signaling Pathway by Enteropathogenic and Enterohemorrhagic *Escherichia coli*

**DOI:** 10.3389/fcimb.2016.00089

**Published:** 2016-08-26

**Authors:** Hilo Yen, Masaki Karino, Toru Tobe

**Affiliations:** Department of Biomedical Informatics, Graduate School of Medicine, Osaka UniversityOsaka, Japan

**Keywords:** enteropathogenic *Escherichia coli*, enterophenorrhagic *Escherichia coli*, type 3 secretion system, effectors, inflammasome

## Abstract

Innate immunity is an essential component in the protection of a host against pathogens. Enteropathogenic and enterohemorrhagic *Escherichia coli* (EPEC and EHEC, respectively) are known to modulate the innate immune responses of infected cells. The interference is dependent on their type III secretion system (T3SS) and T3SS-dependent effector proteins. Furthermore, these cytosolically injected effectors have been demonstrated to engage multiple immune signaling pathways, including the IFN/STAT, MAPK, NF-κB, and inflammasome pathways. In this review, recent work describing the interaction between EPEC/EHEC and the inflammasome pathway will be discussed.

## Introduction

Enteropathogenic and enterohemorrhagic *Escherichia coli* (EPEC and EHEC, respectively) are extracellular pathogens that are transmitted through the ingestion of contaminated food and water. EHEC produces Shiga toxin (Stx), which can cause diseases of hemorrhagic colitis and, in the worst case, hemolytic uremic syndromes. In contrast, EPEC does not produce Stx but can still cause severe gastrointestinal dysfunction, particularly in infants, elders and individuals who are immune incompetent (Nataro and Kaper, 1998; Croxen et al., 2013). EPEC/EHEC targets and intimately attaches to the brush boarder of intestinal epithelial cells to produce characteristic attaching and effacing (A/E) lesions. These lesions are a result of an intense alteration of the host cytoskeleton into a pedestal-like platform. The pathogenicity of EPEC/EHEC depends on the locus of enterocyte effacement (LEE), which encodes type III secretion system (T3SS), a syringe-like apparatus, and secreted virulence factors that are also known as effectors (Moon et al., 1983; McDaniel et al., 1995). Currently, more than 30 different types of effectors have been experimentally verified (Deng et al., 2004; Tobe et al., 2006; Blasche et al., 2014).

Host cells are equipped with pattern recognition receptors (PRRs) that recognize conserved molecules in bacteria. Toll-like receptors (TLRs) are among the well-known PRRs and are located on the surface and endosomal membrane to detect pathogen-associated molecule patterns (PAMPs; Akira and Takeda, 2004; Kawai and Akira, 2011). Moreover, cytosolic nucleotide-binding domain (NBD) and leucine-rich repeat-containing (LRR) proteins (NLR, also known as Nod-like receptor) recognize damage-associated molecule patterns (DAMPs) that arise from cytosolic disturbances or alien substrates, such as membrane dysfunction, pore-forming toxins, bacterial molecules delivered into the cytosol via T3SS or type 4 secretion system (T4SS), and bacterial outer membrane vesicles (Vanaja et al., 2016). The binding of PAMPs or DAMPs activates inflammatory signaling pathways and leads to the production of inflammatory cytokines to further propagate and amplify the immune response (Mogensen, 2009).

To avoid elimination by the host, A/E pathogens have acquired arrays of T3SS-dependent effectors to subvert host-sensing and the activation of inflammatory responses. Specifically, EPEC/EHEC-mediated suppression of the NF-κB pathway and the mechanisms of those prominent NF-κB-suppressive effectors, such as NleB, NleC, NleE, NleH1, and Tir, have recently become clear and have been reviewed elsewhere (Santos and Finlay, 2015). In addition to the NF-κB pathway, an important role of cytosolic NLRs in the sensation of cellular distress caused by pathogens has been increasingly recognized (Moltke et al., 2013; Storek and Monack, 2015). Thus, in this review literature concerning the interactions of NLRs and the inflammasome pathway with EPEC/EHEC will be discussed.

## Inflammasome: sentinel of cellular disturbances

The inflammasome refers to a multimeric protein complex consisting of a sensor, an adaptor, and Caspase-1. The sensor molecule collectively known as NLR is characterized by the presence of a nucleotide-binding domain (NBD) and a leucine-rich repeat (LRR) and can be further categorized into subfamilies with the following distinctive N-terminal effector domains: acidic transactivation domain, pyrin domain, caspase recruitment domain (CARD), and baculoviral inhibitory repeat (BIR)-like domain (Ting et al., 2008; Latz et al., 2013). Upon sensing stimuli, the sensor NLR protein recruits the adaptor protein ASC. ASC is a common component of all of the inflammasome and contains both pyrin and CARD domains that can bridge the NLR molecule and inactive pro-Caspase-1. Subsequently, the incorporation of pro-Caspase-1 into the NLR-ASC complex allows these zymogens to come in close proximity to each other to promote oligomerization and auto-proteolytic cleavage into active Caspase-1 (Yang et al., 1998). Consequently, active Caspase-1 goes on to digest diverse substrates, including pro-IL-1β and pro-IL-18 (Thornberry et al., 1992; Shao et al., 2007; Latz et al., 2013).

**Figure 1:**
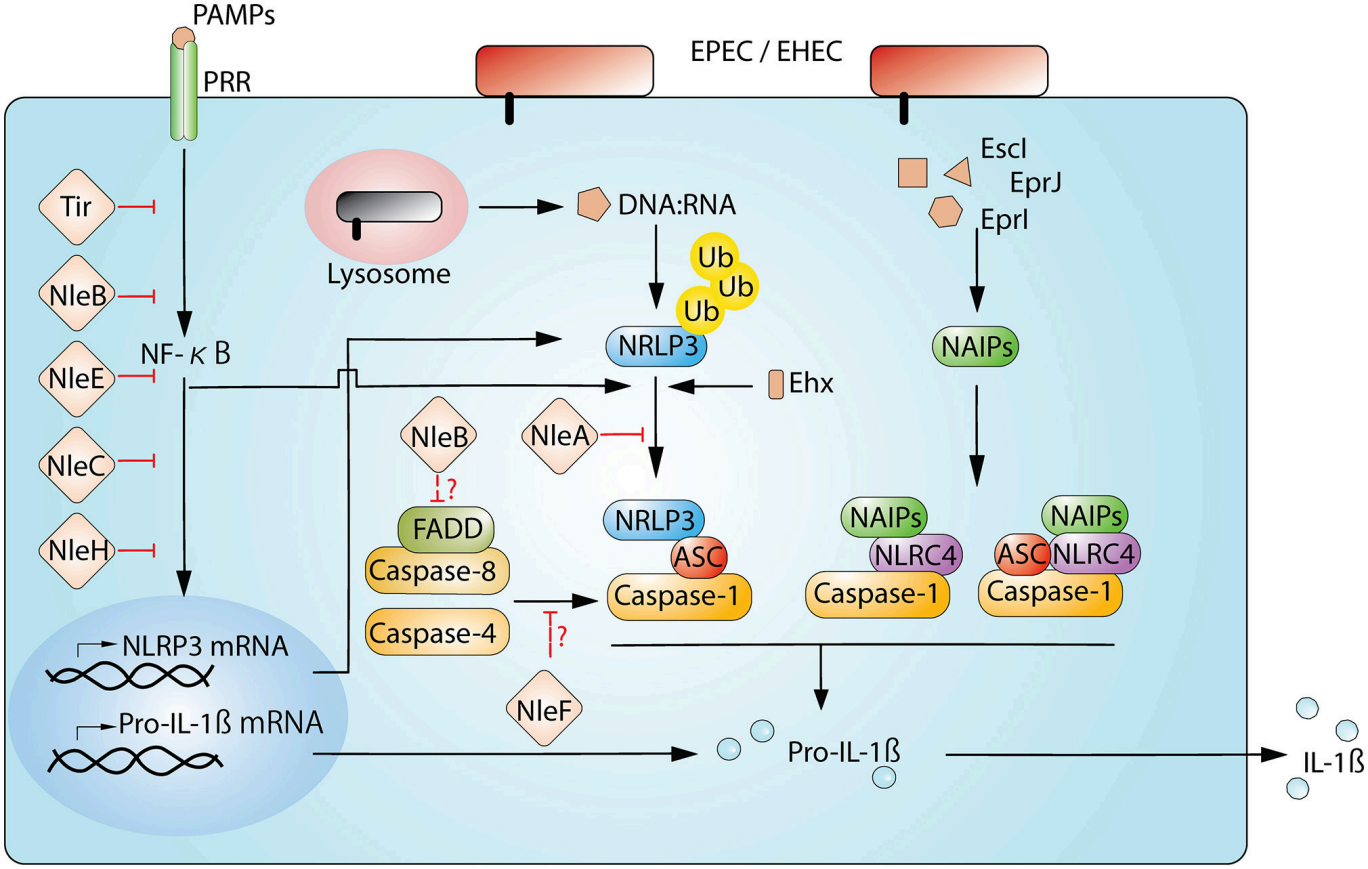
**Interplay between EPEC/EHEC and inflammasome**. Host cells detect and respond to the cellular disturbance from the T3SS-dependent infection of EPEC and EHEC. The sensing of bacterial presence begins when PRRs on the cell membrane recognize PAMPs (for example. LPS by TLR4). This engagement initiates the activation of NF-κB signaling and allows the production of inflammasomal components, including NLRP3 and Pro-IL-1β. Moreover, the detection of PAMPs also triggers the deubiquitination of NLRP3, readying for the sensor protein to assemble the signature complex of NLRP3/ASC/Caspase-1. In the case of NLRP3, the complex formation also requires a secondary stimuli from the infection (for example, the disruption of membrane integrity by Ehx-toxin or the presence of bacterial-derived DNA:RNA hybrids). NLRC4 initiates the complex assembly after Naip proteins binds to respective ligands. Ultimately, pro-IL-1β is processed to a secretion-competent form. EPEC and EHEC use multiple effectors to interfer NF-κB and inflammasome pathways. Currently, only a limited numbers of effectors known to directly target components of the inflammasome complex; they are NleA, NleB, and NleF. NleA directly interacts with NLRP3 to interrupt ubiquitin modification of NLRP3; NleB may negatively influence FADD/Caspase-8, which enhances NLRP3-inflammasome activity; NleF may potentially disrupt NLRP3-inflammasome by inhibiting Caspase-4 and Caspase-8.

One of the most studied NLR proteins is NLRP3. The full activation of the NLRP3-inflammasome pathway requires two steps, i.e., a priming step to activate NF-κB and an activation step to trigger the assembly of the NLRP3/ASC/Caspase-1 complex (Figure 1). Priming is important for the full activation of the NLRP3-inflammasome and begins with the recognition of an NF-κB-activating stimuli, such as PAMP-TLR bindings (the prime example being the binding between LPS and TLR4). This recognition activates the NF-κB-dependent transcription of NLRP3 and IL-1β and the NF-κB-independent de-ubiquitin modification of the NLRP3 protein (Bauernfeind et al., 2009; Juliana et al., 2012; Py et al., 2013). Subsequent to the priming step, the NLRP3-inflammasome assembly is triggered by NLRP3-activating stimuli, which range from metabolic dysregulation (i.e., exposure to uric acid crystals, cholesterol crystals, alum, or extracellular ATP) to infection (i.e., bacterial RNAs, pore-forming toxins, and cytosolically delivered proteins via T3SS/T4SS). This apparent broad responsiveness of NLRP3 suggests that rather than direct recognition of activating agonists, activators may indirectly induce a common downstream perturbation in cellular physiology that leads to the activation of NLRP3. To date, experimentally demonstrated cellular disturbances include potassium efflux (Muñoz-Planillo et al., 2013; Katsnelson et al., 2015), increase in intracellular calcium (Lee et al., 2012; Murakami et al., 2012), the generation of mitochondrial reactive oxygen species (ROS; Zhou et al., 2011), and mitoDNA or cardiolipin (Nakahira et al., 2011; Shimada et al., 2012; Iyer et al., 2013), the release of cathepsin from the disturbed lysosome (Hornung et al., 2008), and the translocation of NLRP3 to the mitochondria (Misawa et al., 2013; Subramanian et al., 2013). However, one needs to be cautious because not every known NLRP3 agonist can induce all of these cellular changes, and the true mechanism(s) of NLRP3 activation remains to be verified. Recently, a non-canonical NLRP3 inflammasome has also been described during infection by enteric pathogens (Kayagaki et al., 2011). This non-canonical NLRP3 pathway involves not only TRL4- and MyD88-mediated NLRP3 upregulation but also the TLR4-TRIP mediated type-I interferon-dependent production of Caspase-11 (Caspase-4 in humans) for maximal Caspase-1 capacity (Wang et al., 1998; Kayagaki et al., 2011; Gurung et al., 2012; Rathinam et al., 2012). This requirement of Caspase-4/-11 for optimal Caspase-1 activation appears to be non-essential for agonists of metabolic dysregulation (Kayagaki et al., 2011).

NLRC4 is another well-studied NLR protein. NLRC4 differs from NLRP3 not only in structure because it has a CARD domain at its N-terminus but also in response; NLRC4 responds to a different and a narrower set of immunogenic agonists. These agonists are mainly the components of T3SS and T4SS (Miao and Warren, 2010; Zhao and Shao, 2015). The CARD motif in NLRC4 allows it to recruit pro-Caspase-1 directly without ASC; however, the incorporation of ASC can maximize the activity of the NLRC4-inflammasome (Mariathasan et al., 2004). Currently there is no evidence demonstrating a direct association of the NLRC4 sensor with its agonists. Rather, studies have confirmed that NLRC4 utilizes co-factors, specifically NLR apoptosis inhibitory protein (NAIP), to distinguish ligands (Tenthorey et al., 2014). Upon recognition of a specific ligand, NAIP then binds to NLRC4 to induce the formation of the NLRC4 inflammasome. In humans, there is one NAIP protein, whereas there are four Naip paralogs that have been confirmed in C57BL/6J mice, including Naip1, Naip2, Naip5, and Naip6. Naip1 shares the greatest homology with the human NAIP and recognizes T3SS needle proteins (Rayamajhi et al., 2013; Yang et al., 2013). Naip2 binds to T3SS rod proteins, and both Naip5 and Naip6 interact with bacterial flagellin (Kofoed and Vance, 2011; Zhao et al., 2011). Notably, not all flagellins or T3SS components can be recognized by the described Naip/NAIP members. For example, while Naip5 and 6 recognize flagellin from *Salmonella* spp., they do not associate with the flagellins of EPEC and EHEC and hence fail to elicit NLRC4 activation (Zhao et al., 2011).

Mice lacking components of the NLRP3- or NLRC4- inflammasome have been found to be more susceptible to infection by various bacterial pathogens (Mariathasan et al., 2005; Lara-Tejero et al., 2006; Raupach et al., 2006; Suzuki et al., 2007). The *in vivo* contribution of host NLRP3 and NLRC4 to act against A/E pathogens have been evaluated using *Citrobacter rodentium* (*C. rodentium*), which is a mouse-adapted A/E pathogen (Alipour et al., 2013). Mice lacking Nlrp3 and/or Nlrc4 carry higher pathogen burdens and exhibit more severe colitis. Dissection of the contributions of the cellular compartments has revealed that cecum-derived E-cadherin+ intestinal epithelial cells (IEC) express high amounts of ASC adaptor, and Nlrp3 and Nlrc4 activation are both critical for protection against *C. rodentium* early in the course of infection (Nordlander et al., 2014; Song-Zhao et al., 2014). However, at later times after infection, the Nlrp3 response mounted by macrophages appears to be indispensable for reducing the severity of the histopathology (Liu et al., 2012).

## Activation of the inflammasome pathway by EPEC/EHEC

Studies of the mechanisms of inflammasome activation during bacterial infection have also provided insight into the components of EPEC/EHEC that are capable of provoking inflammasome pathways. These components include T3SS/*E. coli* type III secretion system 2 (ETT2) components of EPEC and/or EHEC (Miao et al., 2010a; Zhao et al., 2011; Yang et al., 2013), enterohemolysin (Ehx) of EHEC (Zhang X. et al., 2012), and DNA:RNA hybrid molecules (Kailasan Vanaja et al., 2014).

Among the T3SS components, both the inner rod proteins of T3SS and ETT2 (EscI and EprJ, respectively) can activate Caspase-1 via the Naip-Nlrc4 inflammasome. Moreover, the needle protein of ETT2 (EprI) but not that of T3SS (EscF) is able to stimulate IL-1β maturation (Miao et al., 2010b; Zhao et al., 2011; Yang et al., 2013). Interestingly, genomic analysis and functional studies have provided little evidence of functional ETT2 in EHEC or EPEC due to a partial truncation of the encoding loci (Makino et al., 2003; Ren et al., 2004). Although some *E. coli* strains possess an intact ETT2 gene set and may express it, the true physiological relevance of ETT2 components in eliciting Naip/NLCR4-inflammasome activation due to EHEC remains controversial.

In addition to the T3SS and ETT2 components that activate the Naip-Nlrc4 inflammasome, EHEC-specific enterohemolysin (Ehx) has been found to stimulate the maturation of Caspase-1 and IL-1β (Zhang X. et al., 2012). Ehx is a pore-forming toxin encoded on a plasmid that is conserved in most EHEC strains and can cause apoptosis in macrophages and colonic epithelial cells (Schmidt et al., 1995; Menestrina et al., 1996; Fernandez-Prada et al., 1998; Figueiredo et al., 2007). Using siRNA silencing of genes encoding components of the inflammasome complex, Zhang et al. demonstrated that this toxin stimulates the production of mature IL-1β, and this process is partially dependent on NLRP3/ASC/Caspase-1 in human macrophage-like cells (Zhang X. et al., 2012). Given the pore-forming ability of Ehx, this toxin-induced IL-1β production is similar to that of other pore-forming toxins found in different bacterial species that are all known to activate the NRLP3-inflammasome (Gurcel et al., 2006; Mariathasan et al., 2006; Craven et al., 2009). Because these toxins differ in structure, the disruption of the cellular membrane may be the common cause of the stimulation of NLRP3 activity. At the present time, there are no reports on the Ehx-like molecules that cause inflammasome activity in EPEC and *C. rodentium*.

In addition to Ehx, RNA:DNA hybrid molecules of EHEC have also been demonstrated to stimulate NLRP3 inflammasome-dependent IL-1β production (Kailasan Vanaja et al., 2014). The authors of this study demonstrated that, following the phagocytosis of EHEC by mouse bone-marrow-derived macrophages, bacterial RNA:DNA molecules translocate from the Lamp-1-positive lysosome into the cytosolic compartment and colocalize with NLRP3. This colocalization is accompanied with the activation of Caspase-1 and the maturation of IL-1β (Kailasan Vanaja et al., 2014). The sensors of dsDNA (AIM2) and dsRNA (PKR) were found to be dispensable. Currently, the receptor and mechanisms responsible for bringing the DNA:RNA hybrid to NLRP3 remain unknown. This stimulatory property of RNA:DNA hybrids has been confirmed via the use of synthetic RNA:DNA molecules and an RNase H-deficient *E. coli* that accumulates RNA:DNA (Kailasan Vanaja et al., 2014; Vanaja et al., 2015). Therefore, this NLRP3-linked defense mechanism may not be EHEC-specific but rather a general host mechanism for the detection of bacteria in the cytosol.

## Interference with the inflammasome pathway by EPEC/EHEC

The power balance between host immune responses and pathogens dictate the outcome of the infection. In the case of EPEC/EHEC, T3SS-dependent effectors have been demonstrated to modulate various immune signaling pathways (Santos and Finlay, 2015). In addition to the central role of NF-κB in many immune responses, NF-κB is also critical for the activation of the NLRP3-inflammasome because reduced NF-κB activity influences the transcription of NLRP3 and IL-1β (Figure 1; Bauernfeind et al., 2009; Qiao et al., 2012). However, whether the transcription of NLRC4 is similarly regulated by NF-κB is unknown. Thus far, many studies have revealed a great deal of NF-κB-targeting effectors in EPEC/EHEC. Major NF-κB-targeting effectors include NleB, NleC, NleE, NleH1, and Tir (Gao et al., 2009; Nadler et al., 2010; Newton et al., 2010; Royan et al., 2010; Vossenkämper et al., 2010; Yen et al., 2010; Baruch et al., 2011; Mühlen et al., 2011; Ruchaud-Sparagano et al., 2011; Wan et al., 2011; Zhang L. et al., 2012; Gao et al., 2013). Because NF-κB signaling is a prerequisite to the onset of the NLRP3 inflammasome, all of these effectors are expected to contribute to the dampening of NLRP3 activity.

In addition to NF-κB-suppressing effectors, functional studies of other effectors have also yielded evidence that components of the inflammasome are being directly targeted (Table 1; Figure 1). NleA is one such effector that has recently been demonstrated to directly target the NLRP3 protein to prevent Caspase-1 activation (Yen et al., 2015). Initial studies revealed that NleA is a blocker of ER-Golgi transportation (Gruenheid et al., 2004; Kim et al., 2007). Recently, Yen et al. used effector-compound deletion mutants of EPEC to screen for effectors that are able to reduce IL-1β production and isolated NleA (Yen et al., 2015). This reduction in IL-1β due to NleA was not a result of NF-κB inhibition but was rather due to an effect on the assembly of the NLRP3/ASC/Caspase-1 complex. Analysis of the host target revealed that NleA can directly associate with non-ubiquitinated and ubiquitinated NLRP3. Furthermore, this effector has been demonstrated to be capable of interrupting the deubiquitination of NLRP3, which is a pre-requisite to the assembly of the NLRP3/ASC/Caspase-1 complex. How this association negatively affects the de-ubiquitination of NLRP3 is unclear. Thus far, NleA has not been experimentally proven to possess E3-ubiquitin ligase-like activity, and whether this inference with NLRP3 modification is the result of E3 activity is unknown. Another possibility is that because BRCC3 is the sole identified host deubiquitinase of NLRP3 (Py et al., 2013), the association of NleA and NLRP3 may impede the access of BRCC3 to the sensor protein. Therefore, further investigation is required to provide fuller insight into the mechanistic action of NleA.

**Table 1:** **Molecules of EPEC and EHEC that interact with the inflammasome**.

**Molecule**	**EPEC or EHEC specific**	**Inflammasome and/or Sensors**	**Known Function/Inflammasome manipulation**	**References**
EprJ	EHEC	Nlrc4 (murine)	Inner rod protein of ETT2. Induce murine NLRC4 activation. Type of NAIP protein for the recognition is unknown.	Miao et al., 2010a
EscI	Both	Naip2/Nlrc4(murine); NLRC4 (human)	Inner rod protein of T3SS. Induce NLRP4 in a murine Naip2-dependent manner. Recognition by human NAIP is unknown.	Miao et al., 2010b; Zhao et al., 2011
EprI	EHEC	Naip1 (murine); NAIP (human)	A needle protein of ETT2. Induce murine/human NAIP1-dependent activation of NLRC4.	Yang et al., 2013; Zhao and Shao, 2015
NleA	Both ^*^	NLRP3	Inhibit NLRP3-inflammasome formation. Interferes state of NLRP3 ubiquitination.	Yen et al., 2015
NleB	Both ^*^	NLRP3 (?)	Glycosyltransferase; Inhibitor of NF-κB and FADD/Caspase-8 apoptosis; A potential inhibitor of non-Canonical NLRP3 inflammasome by suppressing FADD/Caspase-8.	Gao et al., 2013; Li et al., 2013; Pearson et al., 2013
NleF	Both ^*^	NLRP3 (?)	Inhibitor of Caspase-4,-8, and –9 associated cell death; A pseudosubstrate of Caspase-9; A potential pseudosubstrate to Caspase-4 and –8 by blocking non-canonical NLRP3 inflammasome activity.	Blasche et al., 2013

* NleA, NleB, and NleF are also present in Citrobacter rodentium and rabbit enteric pathogenic Escherichia coli O15:K-:NM (strain RDEC-1).

NleB1 and NleB2 are inhibitors of the Tumor necrosis factor (TNF)-NF-κB signaling axis and are N-acetylglucosamine (GlcNac) transferases (Nadler et al., 2010; Newton et al., 2010; Gao et al., 2013). Moreover, NleB1 can interact with an array of death domain-containing proteins, such as TFNR1-associated death domain (TRADD) and FAS-associated death domain (FADD; Li et al., 2013; Pearson et al., 2013). Specifically, TRADD and FADD are both critical to the TNF-receptor-mediated activation of NF-κB and to the onset of downstream Caspase 8-dependent apoptosis (Micheau and Tschopp, 2003). In the most recent studies, FADD and Caspase-8 have been linked to the regulation of the non-canonical NLRP3 inflammasome. In this process, Caspase-8 augments the NLRP3 inflammasome (Gurung et al., 2014; Antonopoulos et al., 2015; Kang et al., 2015). Thus, in addition to the known NF-κB inhibition, NleB may also provide additional negative regulation of the NLRP3 inflammasome.

NleF is an inhibitor of several Caspase family members, including Caspases-4, 8, and 9 (Blasche et al., 2013). Although the pro-survival effect of NleF is only apparent on ectopic expression, an in-depth analysis of the crystallized structure of the NleF-Caspase-9 protein complex revealed that the effector inserts its carboxyl terminus amino acids, i.e., Gly189, Cys188, Gln187, and Leu186, into the active pockets of the protease. Modification of the carboxyl terminus of NleF by either adding amino acids or deleting the last four amino acids abolishes the activity of NleF. Such biomimicry involving posing as a pseudosubstrate of Caspase enzymes has also been reported in YopM of Yersinia; here, YopM targets Caspase-1 (LaRock and Cookson, 2012). Because Caspase-4 and Caspase-8 both positively participate in non-canonical NLRP3-inflammasome activation, and endogenous bacterially injected NleF has been reported to be low in abundance (Echtenkamp et al., 2008), NleF may serve as a fail-safe mechanism to ensure finer control of the inflammasome pathway.

*In vivo* studies have demonstrated that NleA, NleB, and NleF all are important for colonization and pathology in the *C. rodentium* model of infection (Gruenheid et al., 2004; Kelly et al., 2006; Echtenkamp et al., 2008). Because effectors possess multiple host targets, it is reasonable to speculate that their interference with the inflammasome pathways contributes to the overall bacterial fitness in terms of establishing a successful infection.

## Concluding remarks

IL-1β and related cytokines are critical to host defense in terms of enhancing innate and adaptive immunity against a broad range of pathogens (Netea et al., 2010). In retrospect, this evolutionary selective pressure allows or forces pathogens to develop strategies to counteract the inflammasome pathway, which is an indispensable component of IL-1β production (reviewed elsewhere, Cunha and Zamboni, 2013). Currently, although there is only limited direct evidence of effectors subverting the inflammasome pathway, a close examination of many prior functional studies reveals that EPEC/EHEC, which are similar to other pathogens, have evolved diverse methods that target multiple steps of inflammasome activation (Table 1; Figure 1). Although the reason that A/E pathogens possess molecules that display the opposite effect on the inflammasome activity is unclear, it is likely that the balanced activities by both types of molecules is important for infection. In closing, as many studies have used immune cells to examine the molecular interactions between inflammation and EPEC/EHEC, future studies should utilize intestinal epithelial cells to allow for a deeper appreciation of the complex interaction between the host immune system and A/E pathogens.

## Author contributions

All authors listed, have made substantial, direct and intellectual contribution to the work, and approved it for publication.

## Funding

This work was supported by JSPS KAKENHI Grant Number JP16K19126.

### Conflict of interest statement

The authors declare that the research was conducted in the absence of any commercial or financial relationships that could be construed as a potential conflict of interest.
